# The Research on the Esterification Deacidification of Vacuum Gas Oil by FeZn Bimetal–Organic Frameworks Catalysts

**DOI:** 10.3390/ma18071647

**Published:** 2025-04-03

**Authors:** Bai He, Qing Zhang, Huimin Han, Songshan Jiang, Bo Yu, Shuangkou Chen

**Affiliations:** School of Chemistry and Chemical Engineering, Chongqing University of Science and Technology, Shapingba District, Chongqing 401331, China

**Keywords:** esterification deacidification, bimetal, metal–organic frameworks

## Abstract

The FeZn-MOFs@Al_2_O_3_ catalyst was synthesized under solvothermal conditions. Fourier transform infrared (FT-IR) spectroscopy, X-ray diffraction (XRD), scanning electron microscopy (SEM), X-ray photoelectron spectroscopy (XPS), thermogravimetric analysis (TGA), temperature-programmed desorption of ammonia (NH_3_-TPD), and specific Brunauer–Emmett–Teller (BET) surface area and pore volume were used to systematically investigate the effects of different parameters such as molar ratio of iron to zinc, synthesis temperature, and synthesis time on the properties of the materials. The results showed that the optimum synthesis conditions of FeZn-MOFs@Al_2_O_3_ composites were 140 °C for 1 h, and the optimum molar ratio of Fe^3+^ and Zn^2+^ was 1.3:0.7. Under the aforesaid conditions, FeZn-MOFs@Al_2_O_3_ had the deacidification rate of vacuum gas oil (VGO) up to 96.3%. The optimum esterification parameters were as follows: the amounts of catalyst and ethylene glycol were, respectively, 2.5 wt% and 4.0 wt% of the sample oil, the reaction temperature was 250 °C, and the reaction time was 1 h.

## 1. Introduction

The acidic substance in petroleum is petroleum acid, including 90%, and even more, of naphthenic acids (NAs). The NAs in crude oil lead to deep corrosion and unsafety of oil refining equipment; therefore, it brings an extra cost to the petroleum industry [1]. What is more, the NAs would not only pollute the environment, but also reduce the quality of petroleum products [2]. The NAs are usually present in various kinds of petroleum products, so it is necessary to take measures to remove NAs from oil products. There are many methods to remove the acid in crude oil and oil products, such as the treatment using ionic liquids, catalytic decarboxylation, neutralization or caustic washing, thermal decomposition, physical adsorption, and solvent extraction. However, most of these approaches have drawbacks [3]. Esterification is a promising way to deal with the NAs by reaction into naphthenic acid esters without loss of oil, which overcomes the disadvantages of the traditional deacidification methods for crude oil and petroleum fractions [4], and makes the process suitable for existing refineries. Considering that most of the NAs from crude oils will transfer into VGO in refiners, the deacidification of VGO plays a significant role in the process. In fact, with either crude oil or various distillate oils, the key point is to remove the acid components, especially NAs; therefore, the deacidification technology principles of different crude oils and distillate oil are the same [5]. Some researchers have processed petroleum fractions by catalytic esterification and achieved a deacidification efficiency of up to 95.6%, but the reaction took 6 h [6,7]. It is reported that ZnO- and FeO-doped catalysts have good catalytic esterification activity [8,9], and the catalytic deacidification rate of VGO can be approximately 98.0% using the Fe_2_O_3_-ZnO/Al_2_O_3_ catalyst, but there is the disadvantage that the active metal component load is high and requires many raw materials [10].

Metal–organic frameworks (MOFs) are highly ordered, multidimensional, and reticulate structures made of metal cations and multifunctional organic ligands [11]. The chemical and physical properties of MOFs are rich, and it is easy to improve their performances by modification. Furthermore, the structure of MOFs has a high porosity and a big specific surface area. The cavity of MOFs has open metal active sites, and the cavity shape has excellent diversity and controllability [12,13,14]; therefore, scholars widely use MOFs as catalysts. Although the MOF material itself can be used as a catalyst, its relatively density is small, so it is not easy to separate MOFs from the solid–liquid reaction, especially when the liquid is sticky [15]; thus, researchers usually load MOF materials on other carriers in order to get efficient separation [16,17,18]. Among all kinds of catalyst carriers, Al_2_O_3_ has excellent chemical and thermal stability, special mechanical properties and a cheap price; thus, it is widely used as a catalyst carrier [19,20]. Considering the catalytic esterification activity of Fe-Zn bimetallic catalyst and the excellent attributes of MOFs as catalysts, the FeZn-MOFs/Al_2_O_3_ catalyst was prepared for catalytic esterification decidification of oil product.

## 2. Materials and Methods

### 2.1. Materials

VGO (TAN 2.51 mgKOH/g) was obtained from the China National Offshore Oil Corporation Oil & Gas (Tai Zhou) Petrochemical Co., Ltd. (Taizhou, China) Other chemicals were obtained as follows: ferric nitrate nonahydrate [Fe(NO_3_)_3_ •9H_2_O](analytically pure, Shanghai Macklin Reagent Co., Ltd., Shanghai, China), zinc nitrate hexahydrate [Zn(NO_3_)_2_•6H_2_O] (analytically pure, Shanghai Macklin Reagent Co., Ltd., Shanghai, China), terephthalic acid (H_2_BDC) (analytically pure, Shanghai Macklin Reagent Co., Ltd., Shanghai, China), N, N-dimethylformamide (DMF) (analytically pure, Tianjin Damao Chemical Reagent Factory), anhydrous ethanol (analytically pure, Tianjin Damao Chemical Reagent Factory, Tianjin, China), Al_2_O_3_ (industrially pure, Sinopharm Chemical Reagent Co., Ltd., Shanghai, China).

### 2.2. The Synthesis Methods

**Figure 1:**
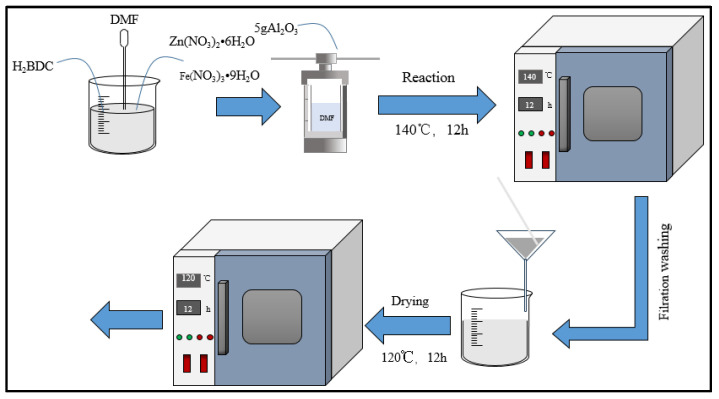
Procedure of catalyst synthesis.

In the solution of DMF, and with the presence of Al_2_O_3_, different proportions of Zn(NO_3_)_2_•6H_2_O, Fe(NO_3_)_3_•9H_2_O, and H_2_BDC were used to synthesize the composite catalyst FeZn-MOFs@Al_2_O_3_; the synthesis path is shown in Figure 1. The mixture was put into the polytetrafluoroethylene reactor and heated at temperatures of 100~160 °C for 6~18 h. When the reaction was completed, the reactor was cooled to room temperature, the lining was taken out, the white sediment products on the bottom were collected, and the sediment was washed three times with DMF and ethanol. The DMF was used to wash away the unreacted solute, and the ethanol was used to wash away the residual DMF. Finally, the products were dried in a vacuum oven at 120 °C for 12 h to obtain the FeZn-MOFs@Al_2_O_3_ catalyst. In order to compare pertinent attributes, the FeZn-MOFs were synthesized by the above procedures.

### 2.3. Charactorization

FT-IR spectroscopy was performed on a Bruker Tensor-27 infrared spectrometer (Bruker, German). XRD patterns were recorded with a Shimadzu XRD-7000 (Kyoto, Japan) using Cu Kα radiation, 2θ = 5°~70°. SEM images were conducted on a JSM-7800F microscope (JEOL Ltd., Akishima, Japan). XPS was investigated with a Thermo Scientific K-Alpha (Thermo Fisher Scientific, Waltham, MA, USA). TGA was performed on a STA449-F3 thermal analysis system (Netzsch, Selb, Germany), under a nitrogen atmosphere, and the heating rate was 10 °C/min. The BET surface area and pore size distribution were verified with N_2_ absorption–desorption isotherms using an ASAP2460 adsorption apparatus (Micromeritics, Norcross, GA, USA), the degassing temperature was 120 °C, and the degassing time was 8 h. NH_3_-TPD results were obtained on a XQ TP5080 temperature-programmed chemical adsorption analyzer (XQ Instrument Co., Ltd., Shanghai, China). The NMR (Nuclear Magnetic Resonance) spectroscopy was conducted with an Agilent DD2 600 MHz (Agilent, Santa Clara, CA, USA), and the solvent was CDCl_3_.

### 2.4. Deacidification Esterification

The catalytic esterification reaction process was as follows: vacuum gas oil, glycol, and the catalyst were put into a three-necked flask, which was fitted with a thermometer and condenser pipe. Then, the reaction system was heated up to the preset temperature and left for some time to react. After the esterification deacidification reaction, the upper refined oil and the catalyst were separated by density difference. The deacidification rate was adopted to evaluate the catalyst effect; the calculation method of the deacidification rate is as follows:

Deacidification rate (%) = (acid value of raw oil-acid value of refined oil)/acid value of raw oil × 100

## 3. Results

### 3.1. The Effect of Synthesis Parameters on the Deacidification Rate

**Figure 2:**
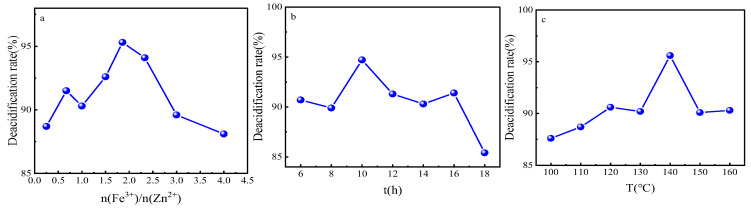
Study on synthesis parameters. (**a**) ratio of Fe^3+^ to Zn^2+^, (**b**) reaction time, (**c**) reaction temperature.

In order to obtain the optimal synthesis parameters for the FeZn-MOFs@Al_2_O_3_ catalyst, the Fe^3+^:Zn^2+^ molar rate, reaction temperature, and synthesis time were investigated; the results are shown in Figure 2. As shown in Figure 2a, with the enlargement of the molar ratio of Fe^3+^:Zn^2+^, the acid removal rate increased and then decreased. When the ratio was 1.86, the catalytic esterification performance was the best, and the deacidification rate was 95.2%. When the ferric nitrate addition was continuously increased, the deacidification began to reduce, probably because that Fe^3+^ replaced some Zn^2+^, which would lead the component and structure change of the catalyst and finally become a disadvantage for the catalytic esterification. Therefore, the molar ratio of Fe^3+^:Zn^2+^ should be 1.86:1. In Figure 2b, it is shown that when the synthesis time was short, the MOFs probably did not complete the forming of the MOF crystals. When the reaction time was too long, the surface of the FeZn-MOFs could break out and influence the surficial metal active sites; thus, the catalytic esterification deacidification effect reduced, so the best synthesis time should be 10 h. In Figure 2c, the deacidification rate reached up to 95.6% with the corresponding temperature 140 °C, then began to decrease with the increased temperature. So the best synthesis temperature should be 140 °C.

Under above optimized synthesis conditions—molar ratio of Fe^3+^/Zn^2+^ = 1.3:0.7, temperature of 140 °C, and time of 10 h—the FeZn-MOFs and FeZn-MOFs@Al_2_O_3_ catalysts were characterized and analyzed as follows.

### 3.2. The Features of the Catalysts

#### 3.2.1. FT-IR Spectroscopy Analysis

**Figure 3:**
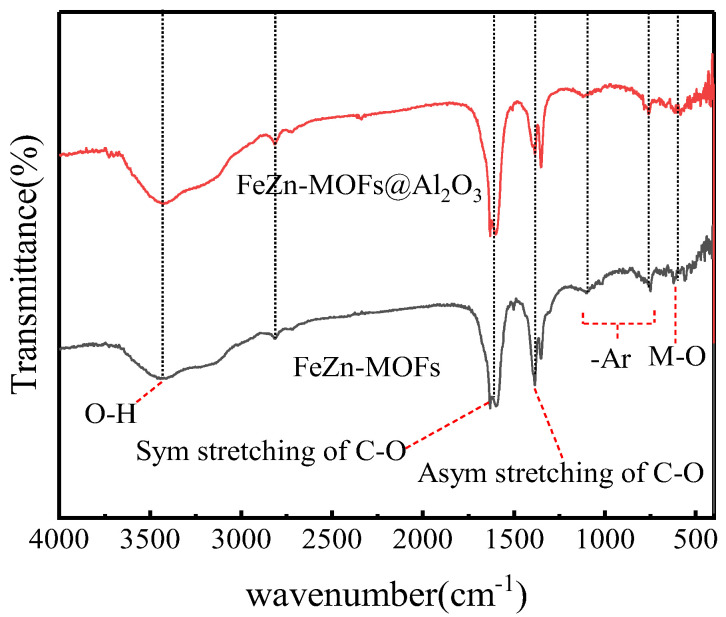
FT-IR spectra of FeZn-MOFs, Zn-MOFs@Al_2_O_3_.

The FT-IR spectroscopy results of FeZn-MOFs and FeZn-MOFs@Al_2_O_3_ are shown in Figure 3; the main absorption bands of the two curves were almost the same. The characteristic infrared band at 554 cm^−1^ is attributed to the stretching vibration of chemical bonds produced by metal ions and oxygen in MOFs [21]. The tensile vibration of carboxyl C-O and O-H were indicated by characteristic infrared bands between 1250 cm^−1^ and 1650 cm^−1^. The symmetric and asymmetric tensile vibration of carboxyl groups in benzoic acid showed two strong characteristic infrared bands at 1380 cm^−1^ and 1610 cm^−1^ [22]. The C-H bond stretching on aromatic benzene rings was indicated by a weak characteristic infrared band at in 2812 cm^−1^. The characteristic infrared band at 3400 cm^−1^ was because of the -OH group of hydrogen bonds between molecules and the O-H group vibration in H_2_O, which could be the ethanol solvent residual and the adsorbed water molecules in the MOF pores [23].

#### 3.2.2. XRD Analysis

**Figure 4:**
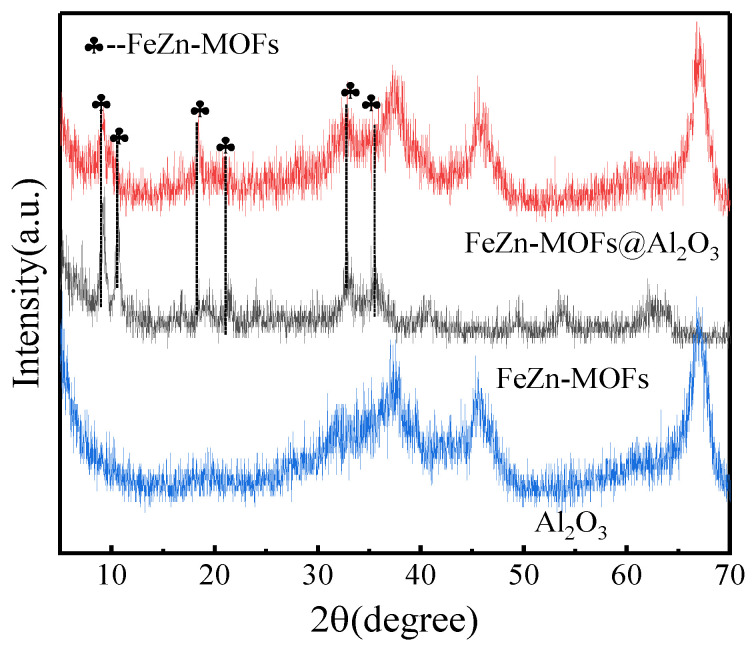
XRD spectra of FeZn-MOFs, FeZn-MOFs@Al_2_O_3_, Al_2_O_3_.

The Al_2_O_3,_ FeZn-MOFs and FeZn-MOFs@Al_2_O_3_ were detected by X-ray diffraction, and the results are shown in Figure 4. The FeZn-MOFs had obvious diffraction peaks at 2ϴ = 9.3°, 16.4°, 19.5°, and 20.5°, and their corresponding crystal planes were (220), (420), (440), and (442) [24], indicating that there was still some zinc-based metal–organic framework crystal in the bimetallic FeZn-MOFs. At the same time, the diffraction peaks at 10.5°, 18.9°, and 22° belonged to Fe-MOF [25], which indicated that either Fe could replace some Zn in the skeleton of Zn-MOF, or there were some Fe-MOFs synthesized and dispersed in FeZn-MOFs@Al_2_O_3_ [26]. In a summary, both FeZn-MOFs and Al_2_O_3_ exist in the FeZn-MOFs@Al_2_O_3_; thus, the proposed catalyst was successfully prepared.

#### 3.2.3. SEM Analysis

**Figure 5:**
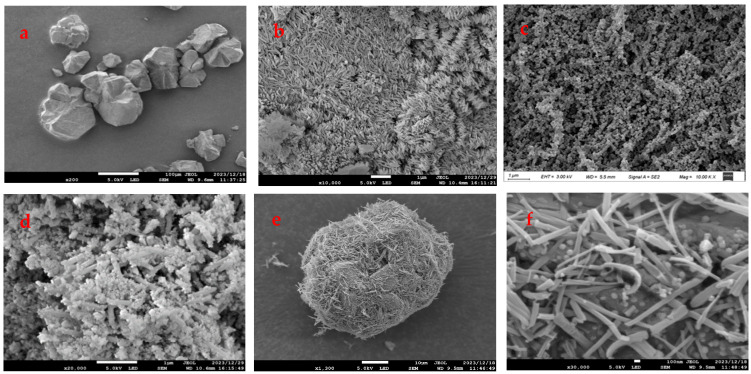
SEM images of the different MOFs: (**a**) Al_2_O_3_, (**b**) Zn-MOFs, (**c**) Fe-MOFs, (**d**) FeZn-MOFs, (**e**,**f**) FeZn-MOFs@Al_2_O_3_.

The microscopic morphology of the materials could be observed by SEM. The SEM results of FeZn-MOFs and FeZn-MOFs@Al_2_O_3_ are shown in Figure 5.

Figure 5a is the image of carrier aluminum; it can be seen that the particles were irregular and the maximum diameter was about 100 μm. Figure 5b shows that the Zn-MOFs were crystal-grained and loose. The surface morphology of Fe-MOFs is shown in Figure 5c; the particle surface seemed to be rod-shaped and porous. In Figure 5d, FeZn-MOFs presented as rod-shaped with irregularly shaped grains, which retained the rod shape of Fe-MOF, but the polyhedral shape in the rod-shaped image was probably because of Zn^2+^ participates, and it indirectly certified that the doping of metal ions could replace some ions of metal clusters and change the crystal structure and morphology. As shown in Figure 5e, FeZn-MOFs were successfully loaded on the surface of aluminum and apparently dispersed. In Figure 5f, the grain and rod-shaped MOFs distributed on alumina completely, and some grain scattered on the surface of the rod-shaped crystals. The MOFs on alumina carrier were dominated by rod crystals, because there was more iron than zinc in the synthesis of FeZn-MOFs; therefore, the bimetallic framework was more inclined to the formation of the Fe-MOF framework. Some research [27] showed that only limited metal ions could replace metal clusters during the bimetallic frameworks synthesis, because the substitution of excessive metal ions would impact crystal generation, and could lead to the changes in the original structure.

#### 3.2.4. XPS Analysis

**Figure 6:**
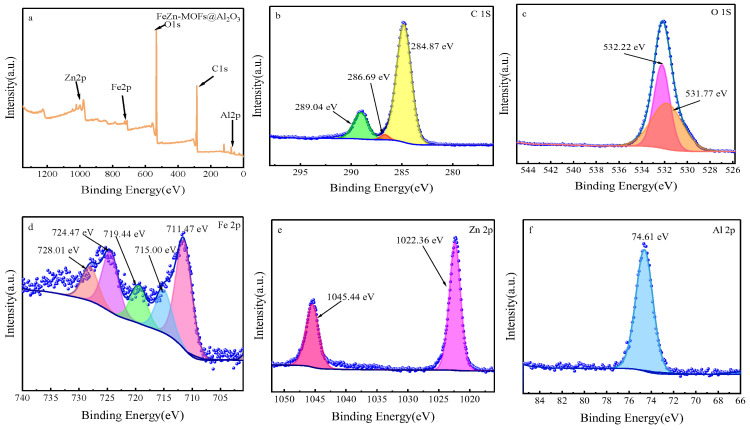
The XPS spectra of FeZn-MOFs@Al_2_O_3_: (**a**) the full spectra of FeZn-MOFs@Al_2_O_3_, (**b**) the C 1s spectra, (**c**) the O 1s spectra, (**d**) the Fe 2p spectra, (**e**) the Zn 2p spectra, (**f**) the Al 2p spectra.

The chemical state and elemental composition of FeZn-MOFs@Al_2_O_3_ catalyst was studied by XPS analysis; the results are shown in Figure 6. Figure 6a is the XPS spectrum of FeZn-MOFs@Al_2_O_3_. It can be seen that the strong peaks at 284 eV, 528 eV, and 1022 eV correspond to C1s, O1s, and Zn 2p [28,29]. The weak peaks at 74 eV and 720 eV correspond to Al 2p and Fe 2p [30,31], which certified that the materials had rich Fe, Zn, O, C, and Al content. Figure 6b is high-resolution XPS spectra of C1s in catalyst, which displayed the characteristic peaks at 284.87 eV, 286.69 Ev, and 289.04 eV that correspond to C-C, C=O, and O-C=O. Figure 6c is the high-resolution XPS spectra of O 1s, which shows two peaks at 531.81 eV and 532.23 eV that correspond to lattice oxygen of MOFs synthesized and the oxygen from carboxylic groups on phthalic acid [32]. The high-resolution XPS spectra of the Fe is displayed in Figure 6d; of the diffraction peaks at 728.01 eV, 724.47 eV, 719.44 eV, 715 eV, and 711.47 eV, wherein the peak at 724.47 eV is ascribed to Fe 2p1/2; the peaks at 711.47 eV and 715.00 eV are attributed to Fe 2p3/2, which proves that the MOFs synthesized had both Fe^2+^and Fe^3+^; and the satellite peaks appear at 728.01 eV and 719.44 eV [33]. In Figure 6e, the peak at 1045.44 eV is assigned to Zn 2p1/2, the peak at 1022.36 eV is related to Zn 2p3/2, and the peak at 1022.36 eV corresponds to Zn-O. In Figure 6f, the peak at 74.61 eV should be assigned to Zn 2p [34].

#### 3.2.5. Thermal Stability Analysis

**Figure 7:**
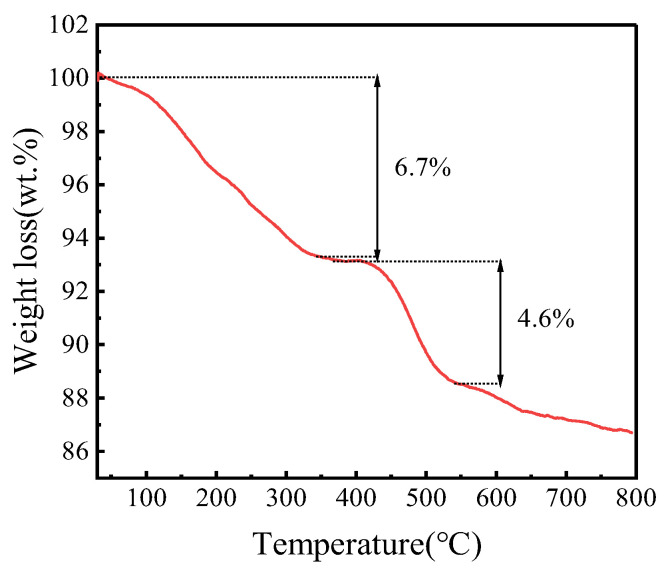
TGA of FeZn-MOFs@Al_2_O_3_.

In order to research the thermal stability of FeZn-MOFs@Al_2_O_3_, a thermogravimetric analysis (TGA) of FeZn-MOFs@Al_2_O_3_ was carried out. It can be seen in Figure 7 that FeZn-MOFs@Al_2_O_3_ has good thermal stability. There are two obvious weight loss stages in the curve. During the first stage, the weight loss is about 6.7%, in which about 4.3% is within the range of 45~185 °C, due to the loss of water adsorbed physically. The slight weight loss at 200~375 °C is about 2.4%, which is probably due to the residual free ligand molecules and solvent, and the carrier alumina reduced the mutual stack of MOFs during its growth, which makes it easier to wash out the residual solvent and guest molecules in the catalyst. The second stage shows the collapse of the crystal skeleton after 375 °C; the metal in the catalyst completely transformed into metallic oxide, the corresponding weight loss is approximately 4.6%, and the surplus weight is 86.8%. The TGA results certified that FeZn-MOFs@Al_2_O_3_ had good thermal stability, it began to decompose quickly until 375 °C, which provided a wide reaction temperature range.

#### 3.2.6. Brunauer–Emmett–Teller (BET) Surface Area and Pore Volume

**Figure 8:**
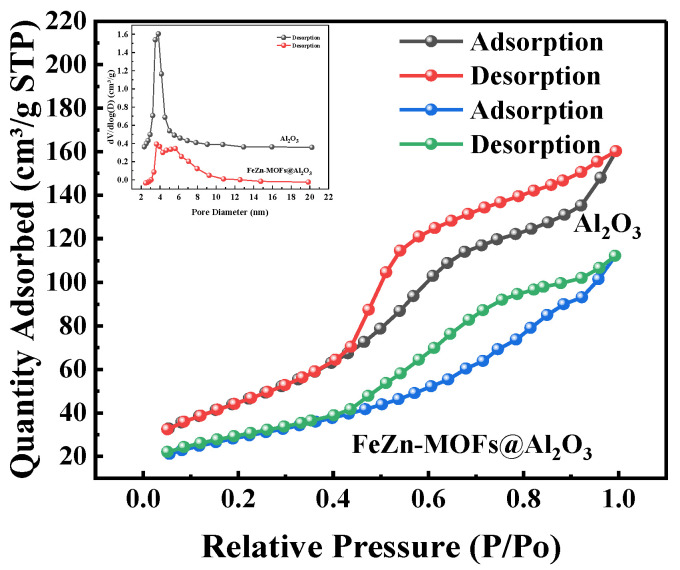
N_2_ adsorption–desorption isotherms and pore size distribution of FeZn-MOFs@Al_2_O_3_.

The N_2_ adsorption–desorption isotherms results and pore size distribution of FeZn-MOFs@Al_2_O_3_ catalyst are shown in Figure 8, and the BET surface areas of the synthesized catalysts are shown in Table 1.

In Figure 8, it can be seen that the FeZn-MOFs@Al_2_O_3_ has adsorption curves of type IV, and the samples had mesoporous structures. In addition, the aperture mainly concentrated within the range of 2.5~10 nm, so FeZn-MOFs@Al_2_O_3_ has mesoporous structure. According to the experimental results in Table 1, it can be concluded that the specific surface area (170.35 cm^2^/g) and pore volume (0.24 cm^3^/g) of the carrier aluminum are both greater than the specific surface area (99.46 cm^2^/g) and pore volume (0.16 cm^3^/g) of the FeZn-MOFs@Al_2_O_3_ catalyst, which may be due to the growth of the MOFs on the alumina.

#### 3.2.7. The Analysis of NH_3_-TPD Catalysts

**Figure 9:**
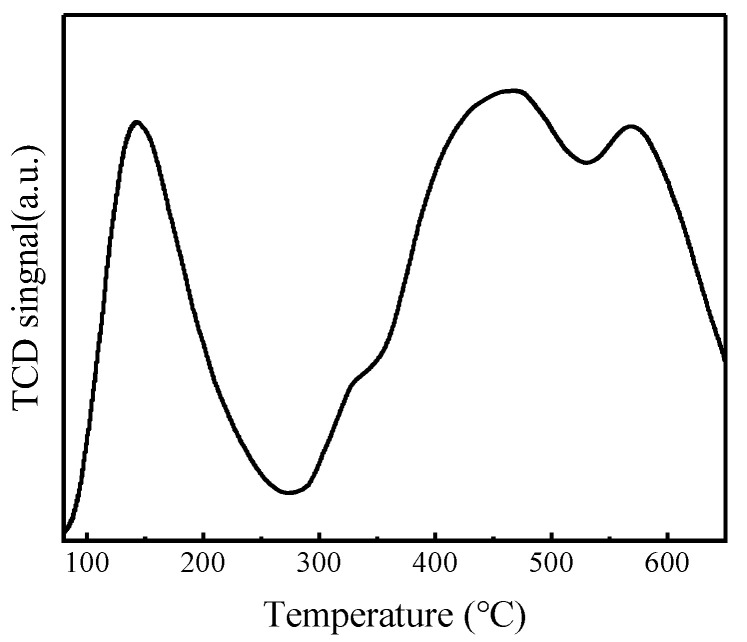
NH_3_-TPD results of FeZn-MOFs@Al_2_O_3_.

NH_3_-TPD characterization results of FeZn-MOFs@Al_2_O_3_ catalyst are shown in Figure 9, and the acid center style and the acid strenth distributing results are listed in Table 2.

According to Figure 9, the NH_3_ desorption curve of FeZn-MOFs@Al_2_O_3_ has four peaks within the temperature range of 100~650 °C. The desorption peak at 143 °C was identified as the weak acid site, the desorption peak at 324 °C revealed the presence of the medium acid strength site, and the desorption peaks at 462 °C and 570 °C showed the strong acid sites. In Table 2, it can be seen that the weak acid amount of the catalyst is 0.11 mmol/g, the medium strong acid amount is 0.03 mmol/g, and the strong acid amount is 0.26 mmol/g. It is reported that the strong acid site benefits the esterification reaction [9]. Therefore, the FeZn-MOFs@Al_2_O_3_ is solid acid catalyst, and the amount of strong acid is more than that of weak acid on the surface of the FeZn-MOFs@Al_2_O_3_, which is good for the esterification deacidification.

### 3.3. Reaction Parameters of Esterification Deacidification

Based on the above results, the esterification deacidification reaction parameters were also investigated, including the dosage of FeZn-MOFs@Al_2_O_3_, the amount of glycol, the reaction temperature, and the time of esterification.

#### 3.3.1. The Catalyst Dosage

**Figure 10:**
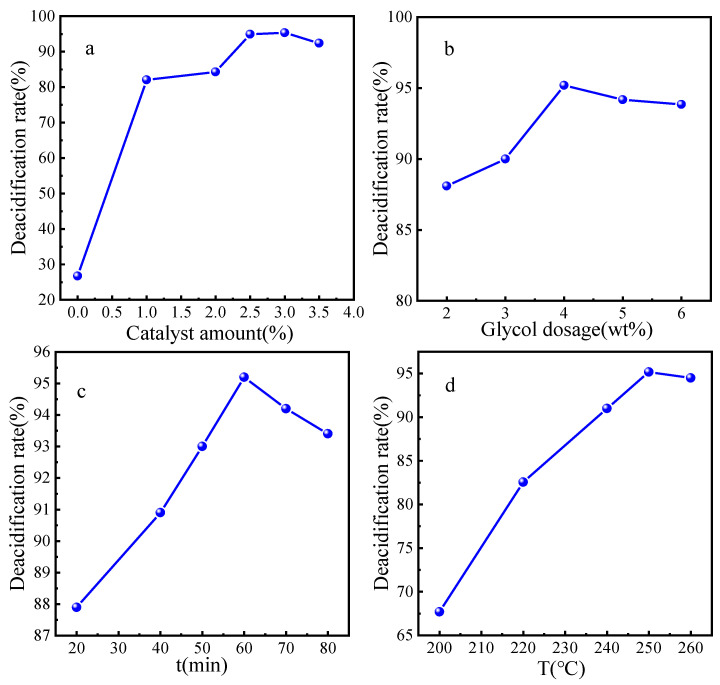
The esterification deacidification reaction parameters: (**a**) Catalyst amount; (**b**) Ethylene glycol amount; (**c**) Esterification time; (**d**) Esterification temperature.

The reaction condition is as follows: 25g VGO, glycol 4.0 wt%, the catalyst dosage 0 wt% to 3.5 wt%, 250 °C for 1 h; the esterification deacidification results are shown in Figure 10a. It can be seen that, if there was no catalyst added, the deacidification rate was just 27.6%, then the deacidification rate increased and decreased with the enlargement of the catalyst. When the catalyst dosage was increased to 3.0 wt%, the esterification deacidification rate reached to the maximum 95.3% because the increase in the total surface area and acid sites of the catalyst with more catalyst, which benefited the deacidification reaction. When the catalyst dosage was more than 3.5 wt%, the deacidification rate began to decrease, probably because too much catalyst leads to the absorption of the reaction mass, which could be adverse to the mass transfer and reaction. Therefore, the optimum amount of catalyst should be 2.5 wt%.

#### 3.3.2. The Amount of Glycol

Theoretically, any alcohols could be the acid removal agents that react with the NAs, but low-boiling-point alcohols such as methanol and ethanol will be almost in a gaseous state at high temperature, and alcohols with super-high boiling points will make them difficult to separate from the oil, especially when the alcohol is overdosed. Therefore, alcohols with proper boling point are very important. Considering the above reasons, glycol was selected for the esterification deacidification in this reasarch.

The reaction condition is as follows: 25 g VGO, catalyst 2.5 wt%, glycol dosage 2.0 wt% to 6.0 wt%, 250 °C for 1 h; the esterification deacidification results are presented in Figure 10b. The acid value of VGO gradually decreased from 0.25 mgKOH/g to 0.11 mgKOH/g, while the amount of glycol was increased to 4.0 wt%; then, additional glycol could not improve the deacidification rate any more. Thus, the proper dosage of the glycol is beneficial to the esterification reaction. However, an overdose of glycol could not only result in side reactions of the glycol, but also lead to the waste of raw materials and heavy post-processing work. Hence, the optimal dosage of glycol dose should be 4.0 wt%.

#### 3.3.3. The Time of Esterification

The esterification time was investigated under the conditions of 25 g VGO, a catalyst dosage of 2.5 wt%, glycol 4.0 wt%, and the reaction temperature of 250 °C; the results are shown in Figure 10c. It is obvious that the deacidification rate increased with longer reaction time: When the reaction time was 20 min, the deacidification rate was 87.9%; when the reaction time was 60 min, the deacidification rate was 95.2%, then the deacidification rate fluctuated slightly. Therefore, if the reaction time was too short, the esterification reaction would not reach equilibrium, and the corresponding deacidification rate would be low. When the reaction time was over the balance time, the glycol would keep volatilizing and the esterification reaction could proceed reversely; then, the deacidification rate decreased accordingly. Moreover, excessive reaction time would consume more unnecessary energy and increase cost, so the proper reaction time should be 60 min.

#### 3.3.4. The Temperature of Esterification

The esterification temperature was investigated under the conditions of 25 g VGO, a catalyst dosage of 2.5 wt%, glycol 4.0 wt%, and the reaction time of 60 min; the results are shown in Figure 10d. It is obvious that the deacidification rate improved gradually with the reaction temperature range 200 °C to 260 °C; the maximum deacidification rate is 95.2% at 250 °C. According to the chemical equilibrium, esterification reaction is an endothermic process; thus, higher temperatures could accelerate the molecular collision and the ester formation. However, the deacidification rate would decrease if the reaction temperature was too high, which could be due to more side reactions and less contact of glycol and NA because of the glycol evaporation. In summary, it is a disadvantage to maximize the deacidification rate and reduce the energy consumption when the reaction temperature is too high or too low. Multiple factors should be comprehensively considered; the optimal reaction temperature should be 250 °C. If the esterification deacidification technology is combined with a refinery unit, no extra heating equipment is needed, because the outlet temperatures of atmospheric furnace and vacuum furnaces are both over 350 °C, which could meet the esterification reaction condition and simplify the process route [3].

### 3.4. Analysis of Refined Oil

In order to validate the deacidification effect of the catalyst and analyze the difference before and after the catalytic esterification reaction of the raw VGO, FT-IR spectroscopy and the ^1^H NMR spectrum were used to compare the product catalyzed by FeZn-MOFs@Al_2_O_3_ and the raw VGO.

#### 3.4.1. FT-IR Spectroscopy

**Figure 11:**
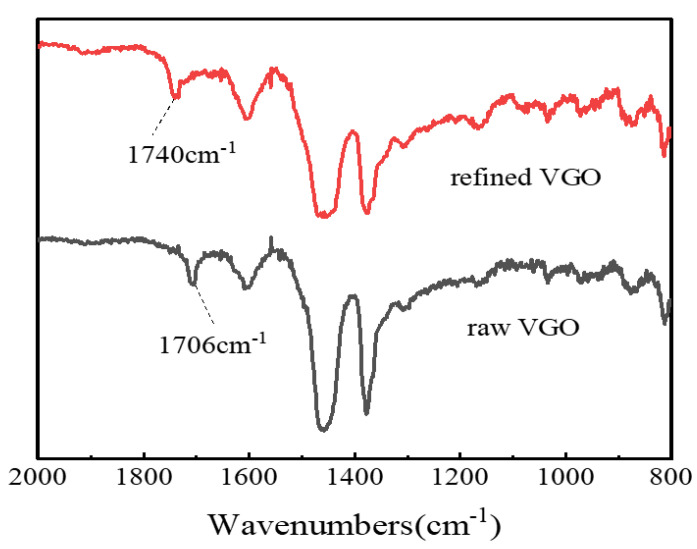
FT-IR analysis of VGO before and after esterification.

The refined VGO obtained under the above optimal catalytic esterification reaction conditions was characterized by FT-IR spectroscopy, and the results are shown in Figure 11. It can be seen that the raw VGO had an obvious carboxylic group (-COOH) characteristic absorption peak at 1706 cm^−1^, but the carboxylic functional group almost disappeared at 1706 cm^−1^ after the esterification reaction, and a new absorption peak appeared at 1740 cm^−1^, which belonged to the ester function (COOC) [35], which certified that the NAs were transformed into esters.

#### 3.4.2. ^1^H-NMR

**Figure 12:**
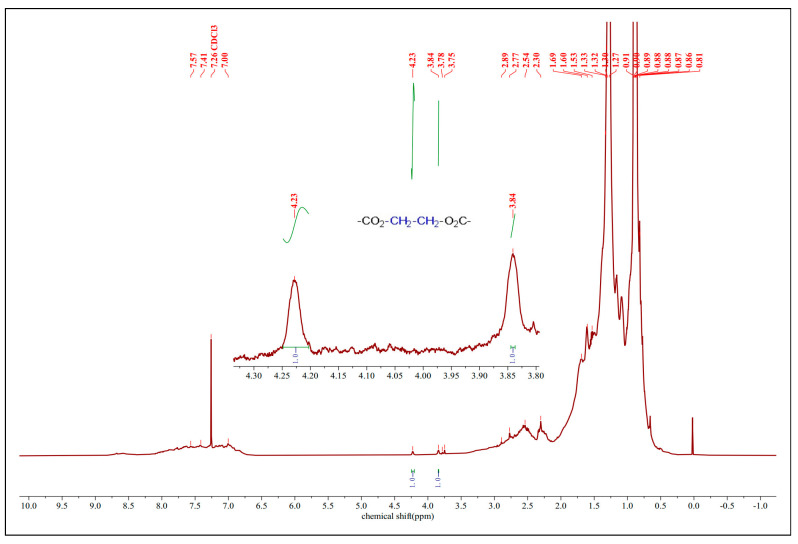
^1^H-NMR of esterified VGO.

The FT-IR spectral data were certified by the ^1^H NMR spectra of the esterified VGO catalyzed by FeZn-MOFs@Al_2_O_3_, as shown in Figure 12. There are two obvious peaks at 3.84 ppm and 4.23 ppm, with the ratio of the integral curve area 1:1, which should belong to the ethylene of the esterified glycol with the NAs. Those two signals could be assigned to the protons of –CO_2_–CH_2_–CH_2_–O_2_C–. The vanishing of the carboxyl proton signal with the appearance of those signals from 3.84 ppm to 4.29 ppm suggest that the esterification of the carboxyl group was accomplished.

#### 3.4.3. ^13^C-NMR

**Figure 13:**
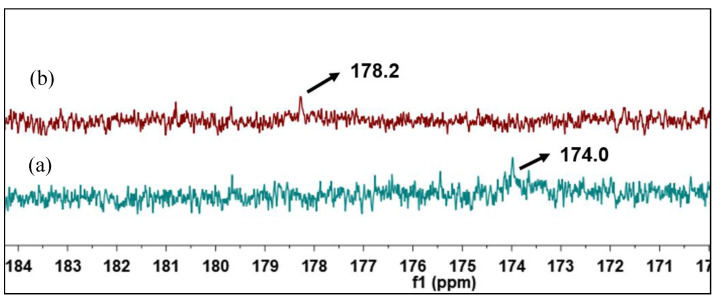
^13^C-NMR spectra of catalytic esterified VGO (**a**) and raw VGO (**b**).

In order to further confirm the transformation of NAs, ^13^C-NMR spectral analysis of raw and catalytic esterified VGO was conducted, and the results are shown in Figure 13. As for raw VGO, the ^13^C NMR chemical shift of carbonyl carbon in NAs was at 178.2 ppm, and there was no esterfication signal at 174.0 ppm. After the raw VGO was catalytically esterified, the acid conponents were turned into esters, there was a new ^13^C NMR chemical shift of ester carbonyl carbon at 174.0, and the orignial chemical shift of NAs carbonyl carbon at 178.2 ppm disappeared, which means that the raw VGO was successfully deacidificated by catalytic esterificaton [35].

## 4. Reaction Mechanism

**Figure 14:**
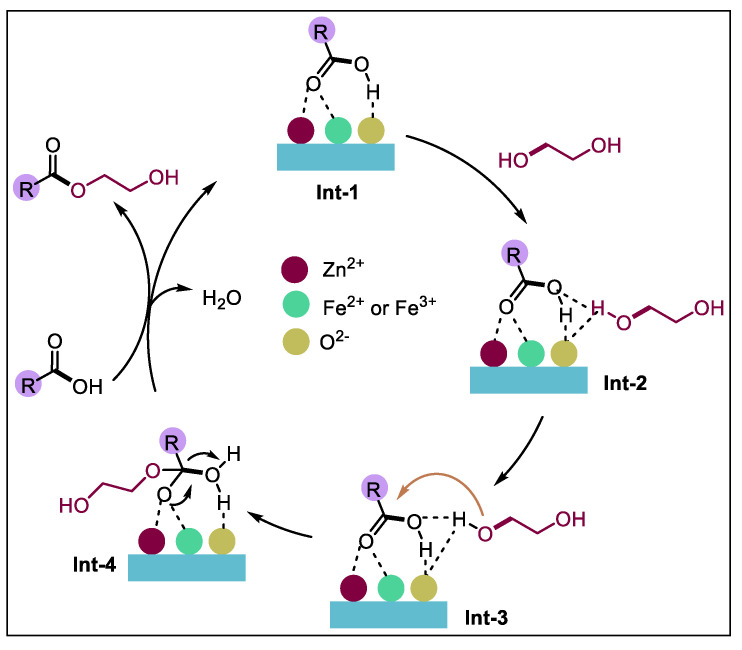
Esterification reaction mechanism of FeZn-MOFs@Al_2_O_3_.

Scholars have conducted much research on esterification and have drawn the conclusion that both Brønsted acid and Lewis acid can play a catalytic role [36,37] in the acid catalytic reaction. The possible catalytic esterification mechanism of NAs by the FeZn-MOFs@Al_2_O_3_ catalysts has been proposed in Figure 14. Initially, the FeZn-MOFs@Al_2_O_3_ catalyst activate NAs through Lewis acid sites (Zn^2^, Fe^2^, Fe^3+^) to generate **Int-1**. Concurrently, hydrogen bonding interactions between the hydroxyl hydrogen atoms of ethylene glycol and the basic O^2−^ centers of the FeZn-MOFs synergistically enhance the nucleophilicity of ethylene glycol, leading to the formation of **Int-2**. Subsequently, the oxygen atom of **Int-2** undergoes nucleophilic attack on the activated NAs, resulting in the generation of **Int-3**. This step was followed by an electron transfer process to form **Int-4**. Ultimately, the desired esterification product and H_2_O were liberated through proton transfer mediated by the NAs, thereby completing the catalytic cycle [38,39,40].

## 5. Conclusions

In this work, FeZn-MOFs@Al_2_O_3_ catalyst was successfully synthesized by a one-step solvothermal method under the optimal preparation conditions: the molar ratio of iron ions to zinc ions of 1.3:0.7, the synthesis temperature of 140 °C, the synthesis time of 10 h; the corresponding catalytic deacidification rate was up to 96.3%. FT-IR spectroscopy, XRD, SEM, XPS, TGA, NH_3_-TPD, and BET surface area and pore volume proved the physical and chemical characteristics of the catalyst. The optimal parameters for esterification were that the catalyst was 2.5 wt% of the VGO sample, glycol 4.0 wt%, a reaction temperature of 250 °C, and a reaction time of 60 min; the highest deacidification rate reached up to 96.3%, which was higher than most of the reported catalytic esterification deacidification rates [41,42]. The FT-IR spectroscopy, ^1^H NMR and ^13^C NMR results proved the deacidification mechanism of the esterification of carboxylic acid groups with glycol. Because of the same deacidification principle, the catalytic esterification deacidification technology in this paper could also be used to effectively process not only VGO but also crude oil and other distillate oils.

## Figures and Tables

**Table 1:** The specific surface area and pore structure parameters of Al_2_O_3_ and FeZn-MOFs@Al_2_O_3_.

Sample	BET Surface (m^2^/g)	Micropore Volume (cm^3^/g)
Al_2_O_3_	170.35	0.24
FeZn-MOFs@Al_2_O_3_	99.46	0.16

**Table 2:** Acid amount of FeZn-MOFs@Al_2_O_3_ catalyst.

Acid Sites (mmol g^−1^)	Weak Acid	Medium-Strong Acid	Strong Acid	Total Acid Content
FeZn-MOFs@Al_2_O_3_	0.11	0.03	0.26	0.40

## Data Availability

The original contributions presented in this study are included in the article. Further inquiries can be directed to the corresponding author.
